# Current Clinical Applications and Future Perspectives of Immune Checkpoint Inhibitors in Non-Hodgkin Lymphoma

**DOI:** 10.1155/2020/9350272

**Published:** 2020-10-29

**Authors:** John Apostolidis, Ayman Sayyed, Mohammed Darweesh, Panayotis Kaloyannidis, Hani Al Hashmi

**Affiliations:** Department of Adult Hematology, King Fahad Specialist Hospital, Dammam, Saudi Arabia

## Abstract

Cancer cells escape immune recognition by exploiting the programmed cell-death protein 1 (PD-1)/programmed cell-death 1 ligand 1 (PD-L1) immune checkpoint axis. Immune checkpoint inhibitors that target PD-1/PD-L1 unleash the properties of effector T cells that are licensed to kill cancer cells. Immune checkpoint blockade has dramatically changed the treatment landscape of many cancers. Following the cancer paradigm, preliminary results of clinical trials in lymphoma have demonstrated that immune checkpoint inhibitors induce remarkable responses in specific subtypes, most notably classical Hodgkin lymphoma and primary mediastinal B-cell lymphoma, while in other subtypes, the results vary considerably, from promising to disappointing. Lymphomas that respond to immune checkpoint inhibitors tend to exhibit tumor cells that reside in a T-cell-rich immune microenvironment and display constitutive transcriptional upregulation of genes that facilitate innate immune resistance, such as structural variations of the PD-L1 locus, collectively referred to as T-cell-inflamed lymphomas, while those lacking such characteristics are referred to as noninflamed lymphomas. This distinction is not necessarily a *sine qua non* of response to immune checkpoint inhibitors, but rather a framework to move the field forward with a more rational approach. In this article, we provide insights on our current understanding of the biological mechanisms of immune checkpoint evasion in specific subtypes of B-cell and T-cell non-Hodgkin lymphomas and summarize the clinical experience of using inhibitors that target immune checkpoints in these subtypes. We also discuss the phenomenon of hyperprogression in T-cell lymphomas, related to the use of such inhibitors when T cells themselves are the target cells, and consider future approaches to refine clinical trials with immune checkpoint inhibitors in non-Hodgkin lymphomas.

## 1. Introduction

The immune system with an orchestrated function of its “machinery” has the capacity to control the immune response to foreign and self-antigens, preventing autoimmunity. This fine tuning is regulated by the immune-checkpoint axis which serves as a “break” to avoid overheating of the “machinery.” The central checkpoint occurs in the lymphoid organs during priming and involves the inhibitory function of the checkpoint molecule cytotoxic T-lymphocyte-associated protein 4 (CTLA-4), which prevents T cells from becoming fully activated upon strong antigen stimulation [1]. The peripheral checkpoint occurs in the peripheral tissues and regulates cytotoxic T-cell (CTL) activation upon T-cell receptor (TCR) binding to the major histocompatibility complex- (MHC-) bound peptide presented on the target cell, hence target-cell lysis. This checkpoint involves the programmed cell-death protein 1 (PD-1) expressed on T cells and its ligands programmed cell-death 1 ligand 1 (PD-L1) and/or PD-L2 which are expressed on the target cells [2, 3]. Binding of PD-L1/PD-L2 to PD-1 receptors leads to inhibition of T-cell function.

Cancer cells and/or nontumor cells from the surrounding microenvironment (ME) commonly overexpress these inhibitory molecules, evading T-cell recognition [4]. The discovery of therapeutic antibodies that block the inhibitory molecules CTLA-4, PD-1, and PD-L1, consequently releasing the “breaks” on these checkpoints, stimulate T cells, unleashing an immune response against cancer cells [5, 6]. Undoubtedly, checkpoint-blockade immunotherapy is one of the most promising advances in cancer treatment [7]. Immune checkpoint inhibitors (ICIs) induce durable clinical responses and are considered part of routine therapy in a growing list of solid tumors [8]. The PD-1/PD-L1 axis also plays an important role for immune evasion of lymphomas [9], most notably in classical Hodgkin lymphoma (cHL) and primary mediastinal B-cell lymphoma (PMBCL), ICIs inducing remarkable response rates in patients with relapsed/refractory (r/r) disease, leading to their approved use in this setting [10–12]. Nevertheless, the role of PD-1/PD-L1 ICIs in various subtypes of non-Hodgkin lymphomas (NHL) is evolving and under investigation. CD47, another immune checkpoint, is a “don't eat me” signal ubiquitously expressed on normal cells and upregulated in various tumors. Macrophages, an essential component of the tumor ME, express signal regulatory protein *α* (SIRP*α*). The CD47/SIRP*α* interaction blocks macrophages from participating in tumor killing [13]. Macrophage checkpoint inhibitors (MCIs) promote phagocytosis of tumor cells and have recently entered clinical development, targeting various tumors, including lymphomas [14].

This review provides an overview of our current understanding of the biological principles and rationale behind immune checkpoint inhibition in NHLs, a summary of published and ongoing clinical trials of ICIs and MCIs in NHLs, and a point of view on the current status and the prospects of moving the field forward in the future. The prognostic relevance of PD-1/PD-L1/PD-L2 as biomarkers of response and the role of the tumor ME in modulating the response to ICIs are discussed in separate articles of this issue.

## 2. Overview of PD-1/PD-L1/2 Expression in NHL, the Cancer Paradigm

PD-1, physiologically expressed by antigen-activated T cells, B cells and natural killer (NK) cells, is considered an exhaustion marker in cancer and chronic viral infections [15]. With regard to PD-1 ligands, PD-L1 (CD274) is expressed by B cells, T cells, and macrophages, while PD-L2 (CD273) is mainly expressed by antigen-presenting cells and epithelial tissues [15]. In many solid tumors, PD-1 is upregulated in the tumor-infiltrating lymphocytes (TILs), whereas its ligands PD-L1 and PD-L2 (less commonly) are expressed by a variety of tumor cells, contributing to the impairment in antitumor immunity [8]. Landmark studies in solid tumors have demonstrated that there are two general mechanisms of PD-L1 upregulation in cancer. The first involves constitutive oncogenic signaling, such as via activation of the AKT pathway or gene amplification upregulated PD-L1 expression on tumor cells, independently of the inflammatory signal in the tumor ME, and referred to as innate immune resistance [16]. The second mechanism is induction of PD-L1 expression, not constitutively, but rather in response to inflammatory cytokines, in particular, interferon-*γ* [17], representing an adaptation of tumor cells to a “hostile” inflammatory ME, referred to as immune adaptive resistance [16]. Thus, the concept of tumors that display a T-cell-inflamed or T-cell-noninflamed phenotype has been introduced [8, 18]; however, these phenotypes are not mutually exclusive and may coexist in the same tumor ME. The T-cell-inflamed phenotype is characterized by the morphologic presence of a preexisting T-cell immune cell infiltrate in the tumor that is negatively regulated by PD-1/PD-L1-mediated adaptive resistance [19] and enriched for response to ICIs through reinvigoration of these preexisting effector antitumor T cells [20], although recruitment of novel T cells that enter the tumor and trigger an effector immune response can also occur [21].

In a similar manner, stratifying lymphomas into inflamed or noninflamed based on the underlying patterns of tumor immunobiology is a rational approach since it provides the means of selecting patients that may benefit from a more personalized approach, incorporating ICIs in the treatment algorithm of disease entities that are sensitive to PD-1/PD-L1 therapy [22]. Interestingly, although interferon-*γ*-mediated upregulation of PD-L1 is a common mechanism of immune adaptive resistance in cancer, upregulation of PD-L1 in lymphomas is frequently driven constitutively by genomic alterations, including structural variation (SVs) in the chromosome region 9p24.1 [23–28], the gene loci where the immunoregulatory genes PD-L1 and PD-L2 reside. The prototype lymphoma that facilitates this concept is cHL, which has an extensive but ineffective immune surveillance ME, crippled by the abundant expression of PD-L1 and PD-L2 on Reed-Sternberg cells, acquired by recurrent copy gains of chromosome region 9p24.1 [23, 24]. These copy gains often include the JAK2 gene locus, resulting in increased JAK/STAT signaling that acts as a promoter for further PD-L1 expression [23]. Data generated from genomic studies and observations in unselected patients from clinical studies of ICIs in NHL, mainly of the large B-cell subtype, suggest that lymphomas with a T-cell-inflamed phenotype collectively share common characteristics, including a T-cell immune surveillance infiltrate [29], genomic alterations that drive overexpression of PD-L1 [23, 26–28, 30, 31], and cell-intrinsic NF-*κΒ* activation [32–34]. Moreover, these T-cell-inflamed lymphomas tend to respond to treatment with ICIs [10–12, 28]. In this review, specific histologic subtypes of NHL will be addressed for the biology of their immune environment, accounting for characteristics that associate with an inflamed phenotype, followed by results of clinical studies in these subtypes and a point of view on current and future directions in these disease entities (summarized in Tables 1 and 2).

## 3. Aggressive B-Cell Lymphomas

### 3.1. Diffuse Large B-Cell Lymphoma (DLBCL)

#### 3.1.1. Biology

DLBCL is currently classified according to cell of origin into two distinct subtypes, the germinal center B-cell-like (GCB) and non-GCB or activated B-cell-like (ABC) [35]. When treated with standard immunochemotherapy, patients with DLBCL of the non-GCB subtype have inferior outcomes compared to those of patients with the GCB subtype [36]. PD-L1 protein expression on tumor cells of Epstein-Barr virus- (EBV)-negative DLBCL is detected in 11%-16% of cases and is consistently higher in the non-GCB subtype of DLBCL [37–39]. More importantly, at the transcriptional level, PD-L1/PD-L2 structural variations (SVs) are found in 20-25% of DLBCL [26, 28], while recurrent translocations between PD-L1/PD-L2 and the IGH locus are also detected and considered a genetic mechanism of PD-L1 (but not PD-L2) overexpression [26]. PD-L1/PD-L2 SVs are associated with upregulation of the NF-*κ*B signaling pathway [28], more common in the non-GCB subtype of DLBCL [26, 28], associated with inferior progression-free survival (PFS) and overall survival (OS), and in the r/r setting tend to respond to anti-PD-1 ICI treatment [28]. With regard to PD-1 protein expression, it is almost exclusively detected on cells of the tumor ME compartment, primarily on TILs, which are significantly higher in the GCB subtype and inversely correlate with the number of PD-L1-positive tumor and ME cells [39].

#### 3.1.2. Clinical Experience

Response rates of DLBCL to single-agent ICIs are generally disappointing. The anti-CTLA-4 antibody ipilimumab as a single agent demonstrates modest activity in patients with r/r B-cell NHL. In a phase 1 study including 18 patients of which only 3 had r/r DLBCL, ORR was only 11%, but interestingly, these few responses were durable, including one in a patient with DLBCL [40]. In a phase 1b study of the anti-PD1 antibody nivolumab in patients with r/r hematologic malignancies, the objective response rate (ORR) in a small number of patients with r/r DLBCL was 36% [41]. In a phase 2 study of nivolumab in patients with r/r DLBCL post autologous stem cell transplantation (ASCT), the reported ORR was 10% and only 3% in ASCT-ineligible patients [42]. Amplifications of 9p.24.1 were detected with fluorescent in situ hybridization (FISH) in 3% of patients. The anti-PD1 antibody pembrolizumab was evaluated as a single agent in a limited number of patients with r/r DLBCL following progression after treatment with anti-CD19 CAR T-cell therapy, yielding an ORR of 25% [43]. More promising results of anti-PD1 ICI monotherapy in r/r DLBCL are derived from the phase 1b KEYNOTE-013 study (NCT01953692). Although the ORR to pembrolizumab in this cohort was 13.8% (4 of 29 patients), interestingly, 2 of 3 patients with PD-L1 SVs had a clinical response, compared to only 2 of 26 patients with no PD-L1 SVs [28]. The presence of PD-L1 SVs was associated significantly with response to pembrolizumab (*P* = 0.005), suggesting that a more personalized treatment approach for patients with lymphomas and inflamed characteristics would be more rational. This concept is tested in an ongoing trial assessing the activity of pembrolizumab in patients with r/r DLBCL and genetic alterations of PD-L1 determined by FISH (Table 3). Durvalumab, an ICI that blocks PD-L1, has also been evaluated as monotherapy or in combination therapy in r/r DLBCL in the phase 1/2 Fusion NHL-001 trial, with no responses in 10 patients treated in the monotherapy arm [44].

The disappointing results of anti-PD-1 and anti-PD-L1 ICIs as monotherapy in unselected patients with r/r DLBCL have led to a plethora of trials evaluating combination therapies in the r/r and upfront setting, in the hope of discovering synergistic effects. Trials with ≥25 enrolled patients are reviewed, while ongoing clinical trials are listed in Table 3. The combination of anti-CTLA-4 with anti-PD1 ICIs has demonstrated superior outcomes as compared to ICI monotherapy in a variety of solid tumors and, consequently, received many approved indications. Therefore, the synergistic or additive effect of combining the ICIs nivolumab and ipilimumab was tested as a hypothesis in patients with r/r lymphoid malignancies, as part of the multicohort phase 1b CheckMate 039 study [45]. The study also included a cohort of patients treated with the combination of nivolumab with lirilumab, an antibody that targets the killer cell receptor immunoglobulin-like receptor (KIR) [46]. KIRs are expressed on NK cells, the principle effector cells of the innate immune system. KIRs interact with HLA molecules and regulate self-tolerance. Disruption of these balancing signals with anti-KIR antibodies, such as lirilumab, can lead to loss of self-recognition (KIR mismatch) and may exert antitumor activity similar to that observed after haploidentical allogeneic stem cell transplantation [47]. The results were discouraging, with ORR and CR rates of 18% and 9%, respectively, for patients with r/r DLBCL treated with the combination of nivolumab and ipilimumab, and likewise an ORR and CR of 12% and 0%, respectively, for patients treated with the combination of nivolumab and lirilumab [45]. The combination of pembrolizumab plus the Bruton tyrosine kinase (BTK) inhibitor acalabrutinib was evaluated in 61 patients with r/r DLBCL with ≥1 prior therapy. The ORR was 26%, and the median duration of response (DOR) was 6.9 months [48]. These results do not differ from the 24% ORR achieved in r/r DLBCL with single-agent acalabrutinib [49]. In a similar phase 1b/2 study in patients with r/r DLBCL or FL, the combination of durvalumab plus the BTK inhibitor ibrutinib induced an ORR of 13% in patients with the GCB subtype and 38% with the non-GCB subtype of DLBCL and 12-month PFS of 12.5% in the GCB and 26.7% in the non-GCB subtype [50], results similar to those observed with the single-agent ibrutinib in a similar setting [51]. In the phase 1b KEYNOTE-155 study, in 38 patients with r/r DLBCL who received ≥2 prior therapies, the combination of pembrolizumab plus dinaciclib, a cyclin-dependent kinase-9 inhibitor, induced an ORR of 18% and a median DOR of 4.9 months [52].

The combination of standard of care R-CHOP with ICIs in previously untreated patients with DLBCL has been evaluated in 3 clinical studies. Final results of a phase 1 study of pembrolizumab plus R-CHOP in 30 patients with previously untreated DLBCL induced an ORR of 90%, including 77% CRs, and with no safety issues [53]. The 2-year PFS was 83%, similar for both GCB and non-GCB subtypes. In an ongoing study, the combination of R-CHOP (GCB subtype) plus durvalumab or lenalidomide plus R-CHOP (R^2^-CHOP) (non-GCB subtype) followed by durvalumab consolidation for up to 12 months is under evaluation in patients with previously untreated DLBCL and high-risk features, mostly with double-/triple-hit lymphoma [54]. At the time of publication, the combinations had an acceptable safety profile and induced an end-of-induction CR rate of 54% for the 30 patients of the GCB subtype. Finally, a phase 1/2 study of R-CHOP plus atezolizumab followed by consolidation atezolizumab induced a CR rate of 77.5% among 40 evaluable patients with untreated DLBCL, with 75% PFS and 86% OS rates at 24 months [55]. Of note, adverse events led to discontinuation of treatment in 36% of patients.

As immune modifying agents, ICIs are currently been assessed in combination with anti-CD19 chimeric antigen receptor (CAR) T-cell therapy, in an effort to enhance the response and durability seen with personalized T-cell therapy. Preliminary results of the ongoing phase 1/2 PLATFORM study evaluating the safety and efficacy of the anti-CD19 CAR T-cell product lisocabtagene maraleucel (liso-cel) in combination with durvalumab in patients with r/r DLBCL demonstrated that the combination has an acceptable safety profile, and the best ORR in a small number of patients was 91% with 64% of patients achieving a CR [56]. The primary analysis of the ZUMA-6 trial, evaluating axicabtagene ciloleucel (axi-cel) plus atezolizumab in 28 patients with r/r DLBCL, showed that the combination had a manageable safety profile, with a best ORR of 75% (45% CR rate) [57], which is similar to that achieved in patients treated with axi-cel alone [58].

Finally, the first-in-class anti-CD47 antibody magrolimab (HU5F9-G4), a MCI, has shown more encouraging results in patients with r/r DLBCL. Interim results of a phase 1/2 trial of magrolimab in combination with rituximab in 46 patients with r/r DLBCL induced an ORR of 39% and complete remission (CR) rate of 20%, and at a median follow-up of 12 months, the median DOR had not been reached (range, 2.4–20+ months) [59].

### 3.2. PMBCL

#### 3.2.1. Biology

PMBCL is a relatively rare subtype of NHL, accounts for 10% of large B-cell lymphomas, and is recognized as a discrete entity by the WHO classification [35]. The disease shares many biological features with cHL, including characteristics of immune evasion due to downregulation of MHC classes I and II and upregulation of programmed death ligands [30, 60–62]. Chromosome 9p24.1 SVs are detected in 63% of PMBCL cases as compared to 38% of cHL cases [23]. Moreover, the copy number of these SVs correlates with PD-L1 and/or PD-L2 expression in primary tumors [25]. Genetic alterations in interferon response genes have also been detected in 52% of cases [63]. PMBCL is highly responsive to anti-PD-1 therapy [12], thus fulfilling characteristics of lymphoma with a T-cell-inflamed phenotype.

#### 3.2.2. Clinical Experience

PMBCL typically presents with an anterior mediastinal mass in young adults, with a higher prevalence in females. Despite high cure rates, achieved with immunochemotherapy with or without consolidative radiotherapy [64–66], 20-30% of patients have r/r disease, commonly widespread and often with central nervous system (CNS) involvement, with poor outcome [67, 68]. Initial results of the phase 1b KEYNOTE-013 study of pembrolizumab in 17 patients with r/r PMBCL demonstrated an ORR of 41% [12]. In a combined report including an update with 21 patients in the phase 1b KEYNOTE-013 study and results of 53 patients from the phase 2 KEYNOTE-170 study, pembrolizumab induced an ORR of 48% (CR 33%) and 45% (CR 13%), respectively [69]. After a median follow-up of 29.1 months in the KEYNOTE-13 study and 12.5 months in the KEYNOTE-170 study, the median DOR was not reached in both cohorts. Of note, none of the patients that achieved CR in the KEYNOTE-13 and KEYNOTE-170 studies had relapsed at the time of the manuscript. In addition, the burden of 9p24.1 SVs was associated with PD-L1 protein expression and PFS, supporting the hypothesis that PMBCL is highly dependent on the PD-1/PD-L1/PDL-2 immune checkpoint pathway. Based on the results of the KEYNOTE-170 study, pembrolizumab has been approved for the treatment of patients with PMBCL who have failed 2 or more prior lines of therapy. It must be emphasized that currently, PMBCL is the only NHL subtype with a commercially approved indication for ICI therapy.

Brentuximab vedotin (BV), an antibody-drug conjugate of monomethyl auristatin E with an anti-CD30 antibody, as monotherapy induces an ORR of 13% in patients with r/r PMBCL [70]. Previous reports have established the safety and efficacy of combining nivolumab plus BV in patients with r/r cHL, suggesting a synergistic effect [71]. Therefore, this hypothesis was tested in the phase 2 CheckMate 436 study, evaluating the combination of nivolumab plus BV in 30 patients with r/r PMBCL [72]. The ORR was 73% with a CR rate of 37%, while median DOR and median PFS had not been reached after a median follow-up of 11 months. In 11 responders, the combination treatment served as a bridging therapy for consolidation with an autologous or allogeneic transplantation.

### 3.3. Primary Central Nervous System Lymphoma (PCNSL) and Primary Testicular Lymphoma (PTL)

#### 3.3.1. Biology

PCNSL and PTL are rare extranodal lymphomas that share common clinicobiological characteristics. These lymphomas arise from previously considered immune sanctuary sites and have inferior responses to treatment [73, 74], are mostly of the non-GCB subtype of DLBCL [75], and have a high prevalence of combined MYD88/CD79B mutations in >70% of cases [76], resulting in constitutive NF-*κΒ* pathway activation. More recently, it was also demonstrated that they have frequent 9p24.1 PD-L1 and PD-L2 copy number alterations which are associated with increased expression of PD-L1 and PD-L2 on the tumor cells [77, 78]. Studies have shown that the tumor microenvironment of PCNSL and PTL is enriched in tumor-infiltrating activated CTLs that express PD-1 and tumor cells that express PD-L1 [79–81] and frequent loss of HLA class I/II and *β*2-microglobulin expression [82, 83]. These collective characteristics suggest that PCNSL and PTL share characteristics of a T-cell-inflamed phenotype.

#### 3.3.2. Clinical Experience

Patients with r/r PCNSL or PTL have a poor prognosis. The T-cell-inflamed tumor microenvironment implicates activity of ICIs in these lymphomas. In a small study of off-label use, 4 patients with r/r PCNCL and 1 patient with CNS recurrence of PTL received the PD-1 ICI nivolumab [84]. All 5 patients had clinical and radiological response; 3 patients remained progression-free at +13 to +17 months. These results, supported by other reports [85, 86], suggest that PCNSL and PTL are sensitive to PD-1 blockade therapy, fulfilling one more characteristic of T-cell-inflamed lymphomas. The immunomodulatory drugs lenalidomide and pomalidomide and the BTK inhibitor ibrutinib have demonstrated notable activity in patients with r/r PCNSL, with radiological responses in at least 50% of patients [87–89]. Ongoing studies are currently evaluating single-agent anti-PD-1 ICIs or in combination with ibrutinib or pomalidomide in r/r PCNSL (Table 3).

### 3.4. EBV-Positive DLBCL and Posttransplant Lymphoproliferative Disorders (PTLD)

#### 3.4.1. Biology

Epstein-Barr virus (EBV) has been implicated in the development of various malignancies, including Burkitt's lymphoma, EBV-positive DLBCL, PTLD, and T-cell/NK lymphoproliferative disorders (discussed further below) [90]. The oncogenic potential of EBV derives from its capacity to exist in a latent state within B cells [91]. EBV-positive DLBCL is an aggressive lymphoma and recognized as a distinct entity in the revised 2016 WHO classification of lymphomas [35]. Most cases have an ABC-like phenotype with upregulation of the NF-*κΒ* pathway [92], characteristically induced by EBV latent membrane protein-1 (LMP1), which explains the rarity of CD79B and MYD88 mutations in these lymphomas [93]. Moreover, 20% of EBV-positive DLBCLs have SVs of PD-L1 and PD-L2 with resulting truncation of the 3′-untranslated regions (3′UTRs) [93] and associated with increased expression of PD-L1 protein on the surface of tumor cells [37]. EBV-positive PTLDs entail a spectrum of lymphoid and plasmacytic proliferations occurring after solid organ or allogeneic stem cell transplantation [94]. The PD-1/PD-L1 axis is also deregulated in PTLDs with PD-L1 expression associated with EBV latency II or III and PD-L1 SVs [95, 96]. It is believed that this tolerogenic immune microenvironment allows EBV-infected cells to evade immune recognition by inducing anergy of anti-EBV CTLs and predisposing to clonal selection and malignant transformation. This also implies that targeting the PD-1/PD-L1 axis with ICIs might be a promising therapeutic option for this lymphoma.

#### 3.4.2. Clinical Experience

The clinical role of ICIs in EBV-positive DLBCL and EBV-related PTLDs is unknown. Case reports/studies of ICIs in EBV-related PTLD post allogeneic stem cell transplantation and other B-cell EBV lymphoproliferations, such as lymphomatoid granulomatosis and hemophagocytic lymphohistiocytosis [97–100], suggest a potential role of ICIs in the treatment of such EBV-related diseases. An interesting ongoing clinical trial is evaluating the role of nivolumab monotherapy or in combination with agents or cellular therapies in EBV-positive lymphomas (Table 3).

### 3.5. Richter Transformation of CLL

#### 3.5.1. Biology

Chronic lymphocytic leukemia (CLL) is characterized by a highly abnormal immune defect in effector T cells of its microenvironment, attributed to the overexpression of PD-L1 which impairs the formation of a functional synapse with the leukemic tumor cells [101], thus limiting their ability to display an antitumor response. Animal models have demonstrated that PD-1 immune evasion in CLL can be reversed with PD-1 ligand antibody [102]. Richter transformation represents transformation of CLL to DLBCL (RT-DLBCL) and is associated with a poor prognosis when the transformation is clonally associated with the underlying CLL (80% of cases) [103]. Interestingly, unlike CLL, tumor cells in RT-DLBCL express high levels of PD-1 [104, 105], which is uncommon in de novo DLBCL. Genetic events associated with this phenomenon have not been elucidated. This observation suggests that PD-1 expression in CLL tumor cells may promote further independence from adaptive immunity and stimulate tumor growth and clonal evolution. PD-1 expression may well serve as a marker of clonal relatedness to CLL and differentiate RT-DLBCL from de novo DLBCL [104]. These observations suggest that the PD-1 blockade may have therapeutic potential in RT-CLL.

#### 3.5.2. Clinical Experience

Clinical studies demonstrate no significant activity of ICIs in r/r CLL, but in contrast to the absence of PD-1 expression in CLL and in concordance with overexpression in RT-DLBCL, ICIs demonstrate notable activity in RT-DLBCL. In a phase 2 study testing the efficacy of pembrolizumab in 25 patients, 16 with relapsed CLL and 9 with RT-DLBCL, the ORR was 0% in CLL and 40% in RT-DLBCL [106]. After a median follow-up of 11 months, the median OS of the RT-DLBCL cohort was 10.7 months. In a study evaluating the combination of nivolumab and ibrutinib in patients with relapsed CLL, the ORR of 33% was similar to that seen with ibrutinib monotherapy, but a promising 65% ORR was induced in 13 of 20 patients with RT-DLBCL [107]. In a small number of patients with BTK-resistant high-risk CLL and RT-DLBCL, the triplet combination of the phosphoinositide 3-kinase (PI3K) inhibitor umbralisib, the anti-CD20 antibody ublituximab, and pembrolizumab induced an ORR of 91% in the CLL cohort and 40% in the RT-DLBCL cohort, with durable responses of 20+ and +12 months in 2 of the 5 treated patients with RT-DLBCL [108]. Overall, these preliminary studies support the further evaluation of PD-1 inhibitors in patients with RT-DLBCL.

## 4. Indolent B Cell Lymphomas

There is limited preclinical and clinical data on the efficacy of ICIs in mantle cell lymphoma, marginal zone lymphomas, and other uncommon indolent subtypes. Therefore, for the purpose of providing a concise discussion of lymphoma subtypes, we will focus only on follicular lymphoma.

### 4.1. Follicular Lymphoma

#### 4.1.1. Biology

FL is the most common indolent lymphoma, accounting for 20% of all new cases of NHL in the western world [35]. In FL, there is a strong crosstalk between the tumor cells and the ME; tumor cells depend on stimuli from the ME milieu to survive and proliferate [29]. Spontaneous regression of disease, frequently observed in patients in whom initial treatment is deferred [109], lends support to the hypothesis that the tumor immune ME may regulate the pace of the malignant process. Moreover, gene expression and subsequently immunohistochemical profiling have shown distinct immune-related signatures associated with an indolent and aggressive form of the disease, highlighting an important interplay between the host immune system and the malignant cells and directly associated with outcome [110, 111].

In contrast to other B-cell lymphoma entities, PD-L1 and PD-L2 are rarely expressed by FL tumor cells [112], and SVs of PD-L1/PD-L2 are infrequent, detected only in 4% of cases [93]; however, PD-1 is abundantly expressed in the ME, though with variable expression patterns [113]. These PD-1-positive cells include TILs, follicular helper T cells (T_FH_), and macrophages [114, 115]. PD-1-positive T cells are more frequently localized in the intrafollicular or perifollicular and less frequently in the interfollicular regions [112]. The composition of the immune infiltrates suggests that FL mounts immune evasion pathways distinct from DLBCL [116]. A number of studies have reported correlation of PD-1/PD-L1/PD-L2 expression with risk of transformation, PFS, and OS, though results are inconsistent [113, 114, 117–119].

#### 4.1.2. Clinical Experience

The results of studies evaluating anti-PD-1 ICIs in FL have been disappointing. In an initial phase 1 study of nivolumab monotherapy in patients with r/r hematologic malignancies, the ORR for 10 patients with r/r FL was 40%, with CR achieved in 1 patient [41]. In the large phase 2 CheckMate 140 study that followed, 92 patients with r/r FL who had failed at least 2 prior lines of therapy received monotherapy with nivolumab. The results were disappointing, with an ORR of only 4% [120]. Similarly, in a phase 2 study in low grade lymphomas, including 18 patients with r/r FL, pembrolizumab monotherapy induced an ORR of 8% [121]. ICIs in combinations with other agents are currently under investigation. In the previously cited multicohort phase 1b CheckMate 039 study, the combination of nivolumab and ipilimumab induced an ORR and CR rate of 20% and 0%, respectively, in patients with r/r FL, while the combination of nivolumab and lirilumab induced an ORR/CR rate of 17% [45]. Interim results of a phase 2 study combining pembrolizumab with rituximab in patients with relapsed FL after ≥1 prior therapy and rituximab-sensitive disease, among 30 evaluable patients, showed an ORR and CR rate of 64% and 48%, respectively, with 60% of patients in ongoing remission after a median follow-up of 11 months [122]. PD-L1 expression in tumor cells was low, while assessment of the baseline tumor immune cell gene signature with NanoString in 12 patients showed a strong correlation of CD8+ effector T cells and induction of CR. These tumor-infiltrating effector T cells have a dysfunctioning immune synapse that can be repaired in vivo with the immunomodulatory drug lenalidomide [123]. In the clinical setting, the chemo-free combination of rituximab plus lenalidomide (R^2^) has demonstrated efficacy in the patients with untreated and r/r FL [124, 125]. Thus, one can hypothesise that lenalidomide might enhance the efficacy of PD-1 inhibitors in FL. Ongoing studies combining ICIs with lenalidomide are addressing this concept (Table 3). In the upfront setting, the phase 2 “1^st^ FLOR” study evaluated priming with single-agent nivolumab followed by combination nivolumab plus rituximab in patients with treatment-naïve FL and an indication for treatment. In an interim analysis of 19 patients (53% high risk FLIPI score), the ORR was 84% (47% CR) [126]. These results should be interpreted with caution since the SAKK 35/98 study of single-agent rituximab followed by maintenance rituximab induced an ORR of 75% and CR rate of 38% in a similar population of treatment-naïve FL patients [127]. Furthermore, response rates to induction therapy with single-agent nivolumab have not been studied, and in addition, a randomized trial with a control arm would be necessary to determine the efficacy of this combination as an upfront treatment strategy in FL.

Studies of PD-L1 inhibitors as monotherapy or in combinations are also under evaluation in patients with FL. In the phase 1/2 Fusion NHL-001 study in patients with r/r DLBCL or FL described previously [44], durvalumab as monotherapy or in combination therapy had limited efficacy, while the R^2^ arm was prematurely closed due to safety concerns raised in another study combining lenalidomide and ICIs in multiple myelomas. In the phase 1/2 study of durvalumab plus ibrutinib, the ORR in patients with r/r FL was 26% [50], comparable with the activity of single-agent ibrutinib in a similar setting [128]. A phase 1b/2 study assessed the safety and efficacy of atezolizumab combined with obinutuzumab and lenalidomide as induction followed by obinutuzumab plus lenalidomide maintenance in patients with r/r FL [129]. In the primary analysis at end-of-induction, the CR rate was 72%, with toxicity consistent with the known profiles of the individual drugs. The authors assert that the efficacy seems higher than that achieved with obinutuzumab plus lenalidomide in the GALEN study [130], but results in a single-arm study should be interpreted with caution considering the caveats entailed when adding an experimental agent to a known effective combination. In the upfront setting, an interim analysis of a phase 1b/2 study of safety and efficacy of induction with obinutuzumab-bendamustine plus atezolizumab followed by maintenance obinutuzumab-atezolizumab in previously untreated patients with FL (20% with a high risk FLIPI score) showed an end-of-induction ORR of 85% and CR rate of 75%, with one treatment-related death [131]. Long-term follow-up data are required to assess the impact of adding atezolizumab to an already active combination in FL.

Finally, the first-in-class anti-CD47 MCI magrolimab (Hu5F9-G4) has been evaluated in combination with rituximab in patients with r/r FL [132]. Updated results of an ongoing phase 1b/2 study demonstrated a promising ORR and CR rate of 66% and 24%, respectively, in the FL cohort [59]. After a median follow-up of 18 months, the median DOR had not been reached. Interestingly, this MCI is well tolerated, induces durable remissions, and is worth further development as a single agent and in combinations in FL.

## 5. Point of View on ICIs in B-Cell NHL

The amazing success story of ICIs in solid tumors has sparked intense interest in studying these immune modifying agents in NHL. Undoubtedly, we have witnessed the impressive impact of anti-PD1 ICIs on outcomes of patients with r/r cHL and PMBCL which has encouraged clinical investigators to pursue clinical trials in many other subtypes of B-cell NHL. Apart from PCNSL, the evidence of meaningful activity in other B-cell NHLs ranges from weak to moderate (Table 1). Following the cancer paradigm, the functional segregation of lymphomas into those with an inflamed versus noninflamed immune phenotype may serve as a predictive biomarker of response to PD-1 ICIs. This holds true for certain subtypes of B-cell NHL, such as PMBCL and PCNSL. These lymphomas have a strong inflamed phenotype which correlates with an exquisite sensitivity to anti-PD1 ICI therapy.

In DLBCL, studies have demonstrated that up to a quarter of cases bear an inflamed phenotype. The poor results of anti-PD-1/anti-PD-L1 ICIs in unselected cases of DLBCL suggest that if there is any benefit in a particular subgroup, this may be lost in the heterogeneity of the disease and the trial design. Therefore, clinical trial design based on common and actionable molecular features of lymphomas is warranted. Towards this direction, following the failure of many studies to capitalize on testing targeted agents based on broad molecular stratifications, such as cell-of-origin in DLBCL, there have been efforts to reclassify lymphomas with the application of next-generation technologies. For DLBCL, although newly proposed molecular classifications by independent research teams overlap in certain subgroups, they do not concur in other subgroups. Nevertheless, they do provide a roadmap for the identification of actionable DLBCLs. Relevant to the purpose of this review, genetic clusters, such as the C1 DLBCL by Chapuy et al. [34] and the so-called BN2 and N1 clusters by Schmitz et al. [33], are associated with immune-related gene signatures that encompass characteristics of inflamed lymphomas. At present, robust platforms to identify such patients are not available but hopefully will be developed in the foreseeable future for incorporation into clinical trial design. In the meantime, an ongoing trial of pembrolizumab in r/r DLBCL with PD-L1 SVs (Table 3) will be important in determining the future of ICIs in DLBCL. Combinations of anti-PD-1/anti-PD-L1 ICIs with other agents or in combination chemotherapy are ongoing (Table 3), although, based on the above caveats of trial design, we believe that there are no grounds for optimism. In addition, there does not seem to be any additional benefit of combining CAR T-cell therapy with anti-PD-L1 ICIs. The first-in-class MCI magrolimab combined with rituximab has shown promising activity in r/r DLBCL and a favorable toxicity profile, justifying its further development. Interestingly, patients who have been previously treated with CAR T cells do not respond to this treatment. At present, there are 2 active large clinical trials evaluating magrolimab in combination with rituximab or the R-GemOx regimen in patients with indolent and aggressive B-cell lymphomas (Table 3), and results are eagerly awaited.

PMBCL, sharing biological and clinical features with cHL, has intense features of an inflamed lymphoma and demonstrates significant and durable responses to single-agent pembrolizumab. Currently, PMBCL is the only NHL with an approved indication for treatment with an ICI. Efficacy is enhanced when pembrolizumab is combined with brentuximab vedotin, suggesting that this combination can serve as an effective and less toxic chemo-free salvage regimen, a bridging therapy to autologous or allogeneic stem cell transplantation or radiotherapy. For those not eligible for transplantation, maintenance therapy with an anti-PD-1 ICI is an appealing option. The combination of anti-PD-1 ICIs with chemotherapy regimens in the upfront setting in patients with high-risk advanced stage disease or at relapse is also an attractive option worth pursuing. In our personal experience single-agent anti-PD-1 ICIs also have activity in patients with CNS disease, inducing durable CRs when combined with cranial radiotherapy in isolated CNS relapse (unpublished data).

PCNSL also has biological features that categorize it as an inflamed lymphoma, reaffirmed by its significant sensitivity to treatment with ICIs. Although data are limited, results look promising. In our personal experience in a handful of cases, durable responses can be achieved with anti-PD1 ICIs in the r/r setting and in patients ineligible for treatment with high-dose methotrexate, particularly when combined with other CNS penetrating novel agents (lenalidomide or ibrutinib) and cranial radiotherapy (unpublished data). Ongoing clinical trials are addressing these questions in patients with r/r disease. Future clinical trials should investigate the role of ICIs in the upfront setting in combination with CNS penetrating agents, most notably in elderly patients or those unfit for aggressive chemotherapy or consolidative autologous stem cell transplantation, which comprise the majority of cases of PCNSL.

Although the immune ME of FL has been extensively studied, suggesting a significant role in sustaining disease activity, it lacks characteristics of an inflamed lymphoma, as currently perceived. Results of clinical trials with ICIs in r/r FL have been disappointing. Single-agent anti-PD1 and anti-PD-L1 ICI ICIs induce single digit responses, indicating that ICIs have no role in the management of FL. Similarly, the simultaneous blockage of two inhibitory signals of the adaptive immune system, CTLA-4 and PD-L1, is not effective. Although combinatory studies of anti-PD-L1 ICIs with a variety of agents with established efficacy show high response rates, results should be interpreted with caution. In contrast to the disappointing responses to ICIs, the MCI magrolimab combined with rituximab has demonstrated promising response rates in r/r FL, and if results are confirmed in the extended ongoing clinical trials, they may pave the way for the approval of this novel immune blockade strategy in FL. Interestingly, MCIs target the nonspecific innate immune response while ICIs bypass the innate component and amplify the adaptive immune response. An effective response of the immune system optimally depends on the dual coordination of the innate/adaptive mechanisms of response. Therefore, it would be interesting to design clinical studies combining ICIs with MCIs to simultaneously unleash the properties of the innate and adaptive immune system and reveal any potential synergism of these checkpoint inhibitors.

## 6. Peripheral T Cell Lymphomas (PTCL)

### 6.1. Biology

PTCLs comprise 5% to 10% of all NHLs [35]; include nodal, extranodal, leukemic, and cutaneous forms; and only recently have been more thoroughly studied [133]. Genetic alterations associated with immune escape via PD-L1 expression have been recently identified in certain subtypes of PTCL, with therapeutic relevance. PTCL, not otherwise specified (NOS) represents a highly heterogeneous group of nodal lymphomas, accounting for a third of the PTCLs in the western world. Signaling pathways that are intrinsically activated and responsible for the expression of PD-L1 are ill defined in this subtype and include SVs of PD-L1 in 15% of EBV positive cases, caused by a truncation of the 3′-untranslated region [93]. PD-L1 is upregulated in anaplastic large cell lymphoma (ALCL), and novel mechanisms of PD-L1 regulation and expression have been discovered in anaplastic lymphoma kinase- (ALK-) positive and ALK-negative subtypes of the disease [134, 135]. Natural killer/T-cell lymphomas (NKTCL) are invariably infected with EBV, and EBV-infected lymphoma cells upregulate PD-L1 expression [37]. Recent data have also revealed distinct molecular subtypes of NKTCL, one of which is of clinical relevance and associates to a subtype with alterations of the immune modulator JAK-STAT mutations/amplification of the 9p24.1/PD-L1/2 locus [136]. Adult T-cell leukemia/lymphoma (ATLL), an aggressive T-cell malignancy caused by human T-cell leukemia virus type 1 (HTLV-1) [137], carries a high mutational burden of genes, including the T-cell receptor, NF-*κ*B, and immune surveillance mechanisms [138]. Aberrant PD-L1 expression is detected in roughly a third of cases of ATLL, owing to SVs commonly disrupting the 3′ region of the PD-L1 gene [138, 139]. Finally, mycosis fungoides (MF) and Sézary syndrome (SS), the most common subtypes of cutaneous T-cell lymphomas (CTCLs), frequently have disruption of the immune evasion PD-1/PD-L1 axis. PD-1 expression is more pronounced in early stages, while PD-L1 expression is strongest in more advanced stages of MF/SS [140]. Furthermore, copy number loss of PD-1 [141] and genomic alterations of PD-L1 have been described in MF/SS [142].

### 6.2. Clinical Experience

Outcomes of patients with PTCL range from unsatisfactory to poor with current therapeutic approaches [143]; therefore, there is an unmet need for effective treatments. The variable upregulation of the PD-1/PD-L1 axis in tumor cells of many PTCL subtypes have led to studies testing the safety and efficacy of ICIs in these lymphomas. Phase 2 studies of single-agent pembrolizumab or nivolumab demonstrated modest activity in a small number of unselected patients with r/r PTCL, inducing ORR of 33% and a number of CRs but with disappointing PFS rates of 2 to 3 months [144, 145]. In one of these studies, there was an alarming dramatic progression of disease within the 1^st^ cycle of treatment, termed “hyperprogression” (discussed further below), in 4 of 12 patients and the study was halted [145]. A phase 1/2 study of pembrolizumab in combination with the histone deacetylase inhibitor (HDACi) romidepsin (an approved treatment of r/r PTCL) in 15 patients with r/r PTCL who had failed to achieve a CR or progressed after ≥1 systemic treatment induced an ORR of 44%, with durable CRs > 10 months in 3 patients [146]. Two patients experienced hyperprogression within the first 10 days of treatment. A small number of patients with ALCL, reported as cases or included in phase 2 studies of unselected patients with PTCL, have been treated with anti-PD1 ICIs [144–149]. With the reservation of potential biased patient selection, results are arguably promising, with notable prolonged CRs independent of the ALK status. Pediatric and adolescent patients with r/r ALCL are the subject of an ongoing clinical trial with the single-agent nivolumab (Table 3).

NKTCL is a specific extranodal PTCL with a very aggressive course. Radiotherapy and asparaginase-based regimens improve outcome and are standard of care [150]. Patients with r/r disease have an extremely poor prognosis, and novel effective agents are an unmet need. A small off-label study of pembrolizumab in patients with r/r NKTCL who had failed an asparaginase-containing regimen demonstrated significant efficacy; out of 7 patients, 5 achieved a CR and maintained their remission after a median follow-up of 6 months [151]. The same authors also assessed the efficacy of off-label low-dose nivolumab in 3 patients with a poor performance status and in a similar setting, demonstrating comparable efficacy and warranting further evaluation of this dosing schedule in patients with NKTCL [152]. In a similar study of low-dose pembrolizumab in 7 patients with r/r disease, 4 patients responded, 2 achieving CR [153]. In the first and largest multicenter phase 2 study to date, sintilimab, an anti-PD-1 ICI approved for r/r cHL in China, was administered to 28 patients with r/r NKTCL who had previously failed an asparaginase-containing regimen. The ORR was 68% and the 1-year OS rate was a promising 82% [154]. It has been recently demonstrated that the presence of mutated PD-L1 is a powerful predictive biomarker of response of NKTCLs to anti-PD1 ICIs [155]. ATLL has clinical subtypes (acute, lymphoma, chronic, and smoldering forms), and its aggressive forms are more common and associated with a poor prognosis [137]. A phase 2 study of nivolumab in patients with ATLL and overexpression of PT-L1 was terminated early when the initial 3 patients developed hyperprogression after a single dose of treatment [156]. Interestingly, this led to the identification of the origin of these malignant cells in the tumor-resident regulatory T cells (Tregs) [157].

Mycosis fungoides (MF) and Sézary syndrome (SS) are the most common subtypes of cutaneous T-cell lymphomas, and treatment options with durable responses in patients with advanced stage disease are limited [158]. A multicenter phase 2 study evaluated the efficacy of pembrolizumab in 28 patients with heavily pretreated advanced stage MF and SS [159]. The ORR was 38%, with 6 of the 9 responding patients demonstrating a 90% or more improvement in skin disease. With a median response follow-up of 12 months, the median DOR was not reached. A cutaneous flare reaction was noticed in 53% of patients with SS, but there were no cases of hyperprogression.

Hyperprogression after treatment with ICIs was first described in patients with solid tumors [160]. Occurring in 5-10% of cases, it represents either the natural history of tumor growth or an immunotherapy-induced acceleration of tumor growth [161]. Hyperprogression should not be confused with the phenomenon of pseudoprogression, an artificial increase in tumor size. Treatment with ICIs induces pseudoprogression by recruiting activated T cells to tumor sites and triggering an inflammatory reaction [162]. As previously cited, it is apparent that the use of anti-PD-1 ICIs in T-cell lymphomas is associated with a risk of hyperprogression, and in some subtypes, such as ATLL, their use is contraindicated. The phenomenon of hyperprogression has been previously recapitulated in a mouse model of T-cell NHL, demonstrating that when the tumor cells are T cells themselves, immunotherapy with ICIs can actively promote tumor progression, implying that in some T-cell malignancies, as in ATLL, PD-1 functions as a tumor suppressor [163, 164]. Finally, there have been a few case reports of secondary T-cell lymphomas developing in patients with solid tumors during treatment with anti-PD1 ICIs [165, 166]. We have also experienced a case of subcutaneous panniculitis-like T-cell lymphoma in a patient with melanoma while on active treatment with an anti-PD-1 ICI (unpublished observation). In one such case, a T-cell clone expanded post-ICI therapy and became a dominant clone leading to development of a T-cell lymphoma following acquisition of a TET2 mutation and loss of PD-1 tumor suppression function [165]. Although rare, these cases suggest that the immune dysmodulating effects of ICIs, typically exemplified with immune-mediated adverse events [167], may predispose or associate with the development of clonal T-cell diseases and warrant further investigation.

## 7. Point of View on ICIs in PTCL

The disproportionate rarity of PTCLs compared to B-cell lymphomas and the heterogeneity and complexity of the disease have limited our understanding of the biology and hampered the development of effective treatments. Although there has been significant progress in deciphering the genomic landscape of the many diseases entailed in the term PTCL, little is known about the mechanisms that underlie the effects of anti-PD-1 ICIs in T-cell-derived tumors, and alarming signals have been associated with their use in certain subtypes.

The most promising results of ICIs in PTCLs undoubtedly originate in the compelling efficacy of anti-PD1 ICIs in r/r NKTCLs, with responses in two-thirds of patients, half of which are CRs. NKTCL has been associated with an inflamed phenotype, corresponding to the TSIM molecular subtype of the disease, and as recently shown, the presence of mutated PD-L1 is highly predictive of response to anti-PD-1 ICIs. Anti-PD-1 ICIs can serve as a bridging therapy to proceed to more intensified therapy or allogeneic stem cell transplantation for those fit and eligible for the procedure, while the identification of patients with the inflamed molecular subtype may serve as a biomarker for a more personalized treatment approach with ICIs in earlier phases of the disease. Clinical trials of the anti-PD1 ICIs nivolumab and sintilimab in combination with asparaginase-gemcitabine-based therapy are under investigation as an upfront therapy in patients with high-risk advanced stage disease (Table 3).

Recent studies have discovered distinct genomic mechanisms of PD-L1 upregulation that coincides with limited but encouraging data that indicate that patients with ALCL may well benefit from treatment with anti-PD1 ICIs. Durable CRs have been reported in a limited number of patients, and an ongoing trial exclusively in young patients with r/r ALCL will hopefully generate positive results. The combination of anti-PD1 ICIs with brentuximab vedotin, an approved indication in r/r ALCL, is an attractive chemo-free option that could be investigated in the appropriate setting. The activity of ICIs in the cutaneous forms of PTCL has been investigated in a limited number of patients with MF and SS, with results suggesting that a third of patients respond to treatment, some of which are durable. Currently, clinical trials are testing the combination of ICIs with approved agents in MF/SS for any additive or synergistic effect, and results will determine their future development in these disease entities.

The disappointing results of anti-PD1 ICIs in PTCL, NOS in combination with the risk of hyperprogression in a third of treated patients led to the termination of studies with ICIs in PTCL, NOS. Similarly, the termination of studies of ICIs in ATLL due to hyperprogression in all initially treated patients sends an alarming message that in some T-cell subtypes, ICIs can be harmful, aggravating disease progression. Therefore, further preclinical and early phase research is warranted before embarking on the next generation of clinical trials in T-cell lymphomas with immune modifying agents.

## 8. Conclusions

The ICIs have become an intense field of clinical research in NHLs. The immune checkpoint axis is transcriptionally and microenvironmentally disrupted in a variety of B-cell and T-cell NHLs. Although many NHL subtypes bear characteristics of inflamed lymphomas, ICIs do not perform well, except for a few subtypes, most notably in PMBCL and NKTCL. In some T-cell lymphomas, ICIs have an opposing effect, leading to hyperprogression. It is apparent that more research is required to better understand how the immune environment functions and the immune mechanisms driving response, resistance, and progression. Furthermore, to overcome the hurdle of the high biological heterogeneity of lymphomas that skews the progress in drug development and personalized therapy, it is anticipated that in the near future with the advances in high-throughput next-generation sequencing (NGS) technologies, a consensus will be achieved for the development of robust NGS platforms for the classification of NHLs, identification of biomarkers relevant to ICI susceptibility, and rational design of targeted clinical trials in the appropriate patients.

## Figures and Tables

**Table 1:** Synopsis of current evidence and evolving role of checkpoint inhibitors in selected subtypes of B-cell NHL.

Histologic subtype	Current level of evidence	
	Alterations associated with an inflamed lymphoma environment	Clinical response to checkpoint inhibitors
DLBCL	*Weak to moderate* (i) Up to 20-25% of DLBCLs have characteristics of an inflamed lymphoma (non‐GCB ≫ GCB) [26, 28, 31]. (ii) Newly proposed molecular classifications of DLBCLs contain genetic clusters (C1 cluster by Chapuy et al. [34] and BN2/N1 clusters by Schmitz et al. [33]) that encompass characteristics of inflamed lymphomas. (iii) Development of robust platforms to identify inflamed cases are warranted.	*ICIs: weak* (i) Disapointing activity of monotherapy with anti-PD1 and anti-PD-L1 ICIs in r/r DLBCL [28, 41–44]. (ii) Significant association of presence of PD-L1 SVs with response to anti-PD1 ICIs [28], although results based on a small number of cases. (iii) Results of an ongoing study of pembrolizumab in patients with r/r disease and PD-L1 SVs (NCT03990961) will determine the future development of anti-PD1 ICIs in subsets of DLBCL. *MCIs: moderate* (i) Magrolimab demonstrates a favorable toxicity profile and notable activity in r/r DLBCL, supporting its further development and investigation in combination with other agents [49].

PMBCL	*Strong* (i) Two-thirds of cases have characteristics of an inflamed lymphoma [23, 30, 31].	*Strong* (i) Currently the only approved lymphoma with indication for treatment with a ICI. (ii) Significant activity and durable remissions with pembrolizumab in r/r PMBCL [69], burden of 9p24.1 SVs associated with PD-L1 expression and improved PFS. (iii) The combination of nivolumab and brentuximab vedotin is highly active in r/r PMBCL [72]. And effective bridging therapy for other consolidative treatments (auto- or allo-SCT) of curative intent. (iv) Clinical trial assessing the addition of anti-PD-1 ICIs to standard regimens in the upfront setting is warranted for patients with high-risk advanced stage disease.

PCNSL and PTL	*Strong* (i) Two-thirds of cases have characteristics of an inflamed lymphoma [76–78, 82, 83].	*Moderate to strong* (i) High clinical and radiological response rates to monotherapy with anti-PD1 ICIs but studied in a limited number of patients [84–86]. (ii) Results of ongoing clinical trials will determine further development.

RT-DLBCL	*Moderate* (i) Strong expression of PD-1 in majority of cases [104].	*Weak to moderate* (i) Noteworthy activity of anti-PD1 ICIs, higher ORRs when combined with ibrutinib [106, 107]. (ii) Ongoing clinical trials of anti-PD1 ICIs with novel agents will determine further development.

FL	*Weak* (i) PD-L1/PD-L2 SVs are rare in FL [93]. (ii) In contrast, abundant expression of PD-1 is found in cells of the immune ME (TILs, TFH cells, and macrophages) [74–76].	*ICIs: weak* (i) Disappointing activity of monotherapy with anti-PD1 or anti-PD-L1 ICIs in r/r FL [41, 44, 121]. (ii) Preliminary results of clinical trials combining ICIs with other agents have not demonstrated any apparent incremental benefit [122, 126, 129, 131]. *MCIs: moderate to strong* (i) Magrolimab demonstrates a favorable toxicity profile and significant activity in r/r FL, supporting its further development and investigation in combination with other agents [59, 132].

Abbreviations: CNS: central nervous system; CR: complete remission; DLBCL: diffuse large B-cell lymphoma; DOR: duration of response; GCB: germinal center B cell; ICI: immune checkpoint inhibitor; MCI: macrophage checkpoint inhibitor; ME: microenvironment; ORR: overall response rate; PCNSL: primary central nervous system lymphoma; PD-1: programmed cell-death protein 1; PD-L1: programmed cell-death 1 ligand 1; PFS: progression-free survival; PMBCL: primary mediastinal B-cell lymphoma; PTL: primary testicular lymphoma; PTLD: posttransplant lymphoproliferative disorder; RT-CLL: Richter's transformation of chronic lymphocytic leukemia; SVs: structural variations; TFH: T follicular helper; TILs: tumor-infiltrating lymphocytes.

**Table 2:** Synopsis of current evidence and evolving role of checkpoint inhibitors in selected subtypes of T-cell NHL.

Histologic subtype	Current level of evidence	
	Alterations associated with an inflamed lymphoma environment	Clinical response to checkpoint inhibitors
PTCL, NOS	*Weak* (i) A small subset of EBV-positive cases bears structural variations of PD-L1/PD-L2 [93].	*Weak: studies with anti-PD-1 ICIs terminated due to cases with hyperprogression* (i) Disappointing ORR and DOR with anti-PD-1 ICIs in r/r disease [144, 145]. (ii) Hyperprogression in a third of cases; studies terminated.

ALCL	*Moderate* (i) PD-L1 upregulation in ALK-positive and ALK-negative cases [134, 135].	*Moderate to strong* (i) Data on a limited number of patients originating from small studies and case reports show impressive responses and durable CRs with anti-PD-1 ICI monotherapy in both ALK-positive and ALK-negative cases [145–149]. (ii) Ongoing clinical trial to determine efficacy in r/r setting and as consolidative therapy in patients achieving CR.

NKTCL	*Moderate to strong* (i) Distinct molecular subtype with inflamed phenotype (PD-L1/PD-L2 SVs and JAK/STAT mutations) [136]. (ii) EBV-upregulated PD-L1 expression [37].	*Strong* (i) High ORR and CR rates to anti-PD-1 ICIs in patients with r/r disease failing prior treatment with asparaginase [151–154]. (ii) Presence of mutated PD-L1 is a powerful predictive biomarker of response to anti-PD1 ICIs [155]. (iii) Ongoing clinical trials in upfront and r/r setting

ATLL	*Moderate* (i) Aberrant PD-L1 expression in one-third of cases [138]. (ii) High mutational burden of TCR and NF-*κ*B genes [139].	*Studies with anti-PD-1 ICIs terminated due to hyperprogression* (i) Hyperprogression developed in first 3 patients after a single dose of anti-PD-1 ICI [156]. (ii) Hyperprogression implies that PD-1 functions as a tumor suppressor in ATLL and other T-cell malignancies [163].

MF and SS	*Moderate* (i) PD-1 expression more pronounced in early stages and PD-L1 in more advanced stages of disease [140]. (ii) Genomic alterations of PD-1 and PD-L1 [141, 142].	*Moderate* (i) Responses to pembrolizumab in over one-third of patients with r/r MF or r/r SS [159]. (ii) Cutaneous flare reaction common in patients with SS; no cases of hyperprogression. (iii) Ongoing studies further assessing role of ICIs in cutaneous T-cell lymphomas.

Abbreviations: ALCL: anaplastic large cell lymphoma; ALK: anaplastic lymphoma kinase; ATLL: adult T-cell leukemia/lymphoma; CR: complete remission; DOR: duration of response; EBV: Epstein-Barr virus; ICI: immune checkpoint inhibitor; IGH: immunoglobulin heavy chain; JAK: Janus kinase; MCI: macrophage checkpoint inhibition; ME: microenvironment; MF: mycosis fungoides; NF-*κΒ*: nuclear factor kappa-light-chain-enhancer of activated B cells; NKTCL: natural killer T-cell lymphoma; ORR: overall response rate; PD-1: programmed cell-death protein 1; PD-L1: programmed cell-death 1 ligand 1; PTCL, NOS: peripheral T-cell lymphoma, not otherwise specified; r/r: relapsed/refractory; SS: Sézary syndrome; STAT: signal transducer and activator of transcription proteins; SVs: structural variations; TCR: T-cell receptor.

**Table 3:** Summary of selected ongoing clinical trials of PD-1/PD-L1 ICIs in NHL.

Disease, phase	Phase	Intervention	ClinicalTrials.gov NCT reference
Aggressive B-cell lymphomas			
DLBCL, r/r	Ib	Pembrolizumab in combination with tisagenlecleucel	NCT03630159
DLBCL and PMBCL, r/r	II	Pembrolizumab in combination with copanlisib	NCT03484819
DLBCL with PD-L1 genetic alterations, r/r	II	Pembrolizumab monotherapy	NCT03990961
Aggressive B-cell lymphoma, untreated	II	Nivolumab in combination with DA-EPOCH-R	NCT03749018
HGBCL with MYC, BCL2, and/or BCL6 rearrangement, untreated	II	Nivolumab as consolidation after DA-EPOCH-R	NCT03620578
DLBCL, untreated	II	Avelumab as induction and maintenance with R-CHOP	NCT03244176
EBV-positive NHL and EBV-positive PTLD, untreated or relapsed	II	Nivolumab monotherapy	NCT03258567
DLBCL, r/r	Ib	Magrolimab (Hu5F9-G4) in combination with rituximab or R-GemOx	NCT02953509
Aggressive B-cell lymphomas, extranodal			
PCNSL or PTL, r/r	II	Nivolumab monotherapy	NCT02857426
CNS lymphoma, r/r	II	Nivolumab in combination with ibrutinib	NCT03770416
PCNSL, relapse after prior 1^st^ line HiDMTX	II	Pembrolizumab monotherapy	NCT02779101
PCNSL, r/r	I	Nivolumab in combination with pomalidomide	NCT03798314
Transformed indolent B-cell lymphoproliferative diseases			
RT-DLBCL or transformed FL	I	Nivolumab in combination with copanlisib	NCT03884998
RT-DLBCL	II	Atezolizumab in combination with obinutuzumab+venetoclax	NCT04082897
Peripheral T-cell lymphomas			
NKTL, untreated or r/r	I	Nivolumab in combination with L-asparaginase/GDP	NCT04230330
NKTCL, untreated	II	Sintilimab in combination with Peg-asparaginase/GemOx	NCT04127227
MF and SS, r/r		Pembrolizumab combined with radiotherapy	NCT03385226
ALCL, r/r	II	Nivolumab as treatment or consolidative immunotherapy	NCT03703050
Cutaneous TCL, r/r	II	Atezolizumab monotherapy	NCT03357224
PTCL or cutaneous TCL, r/r	I/II	Durvalumab±lenalidomide	NCT03011814

Abbreviations: ALCL: anaplastic large cell lymphoma; CNS: central nervous system; DLBCL: diffuse large B-cell lymphoma; EBV: Epstein-Bar virus; FL: follicular lymphoma; HGBCL: high-grade B-cell lymphoma; HiDMTX: high-dose methotrexate; LPD: lymphoproliferative disorders; NHL: non-Hodgkin lymphoma; NKTL: natural killer T-cell lymphoma; PCNSL: primary central nervous system lymphoma; PD-1: programmed cell-death protein 1; PD-L1: programmed cell-death 1 ligand; PMBCL: primary mediastinal B-cell lymphoma; PTCL: peripheral T-cell lymphoma; PTL: primary testicular lymphoma; RT-DLBCL: Richter's transformation of CLL to DLBCL; TCL: T-cell lymphoma.
